# Serum hsa_circ_0000615 is a prognostic biomarker of sorafenib resistance in hepatocellular carcinoma

**DOI:** 10.1002/jcla.24741

**Published:** 2022-10-21

**Authors:** Lunjun Zhang, Tao Xu, Yuanyuan Li, Qing Pang, Xiaolin Ding

**Affiliations:** ^1^ Department of Clinical Laboratory Science The First Affiliated Hospital of Bengbu Medical College Bengbu China; ^2^ Department of Clinical Laboratory, School of Laboratory Medicine Bengbu Medical College Bengbu China; ^3^ The Second Clinical Medical College of Anhui Medical University Hefei China

**Keywords:** hepatocellular carcinoma, hsa_circ_0000615, sorafenib resistance

## Abstract

**Background:**

Circular RNAs (circRNAs) can shape tumor progression and chemoresistance. How specific circRNAs shape hepatocellular carcinoma (HCC) chemoresistance, however, remains to be fully elucidated.

**Methods:**

In total, serum samples were collected from 202 HCC patients that had completed four sorafenib chemotherapy cycles. Serum hsa_circ_0000615 levels in these patients were quantified via quantitative real‐time polymerase chain reaction (qRT‐PCR), with demographic details and survival outcomes being recorded for subsequent analyses.

**Results:**

We found hsa_circ_0000615 to be significantly upregulated in chemoresistant HCC patients relative to chemosensitive patients, with such upregulation being positively correlated with disease stage. Moreover, the area under the curve (AUC) value for hsa_circ_0000615 was moderately good, and high levels of hsa_circ_0000615 expression were associated with shorter overall survival among chemoresistant HCC patients.

**Conclusion:**

Our results highlight hsa_circ_0000615 as a promising driver of sorafenib resistance in HCC patients, highlighting it as a promising target for the treatment of this deadly cancer type.

## Graphical abstract

In this study, we herein found circZNF609 upregulation to be prominent within serum samples from HCC patients, with such upregulation being significantly more pronounced in samples from chemosensitive patients relative to chemoresistant patients. As such, circZNF609 is a promising target that warrants further study in an effort to understand the mechanistic basis for HCC patient chemoresistance.

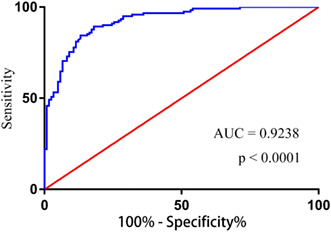

## 1 INTRODUCTION

Hepatocellular carcinoma (HCC) is the leading cause of cancer‐related death worldwide.^1^ In most cases, long‐term sorafenib administration is associated with the onset of chemoresistance. As such, there is a clear need to identify the mechanistic basis for sorafenib resistance and to design novel approaches to effectively treat this cancer type.

Circular RNAs (circRNAs) are a covalently closed looping structure that renders them resistant to degradation and more stable than linear RNAs.^2^, ^3^, ^4^ A growing body of evidence suggests that circRNAs can regulate a range of cancers and other important diseases by influencing cellular proliferation, survival, migration, glucose metabolism, and differentiation.^5^, ^6^, ^7^, ^8^, ^9^ As such, circRNAs offer great promise as diagnostic biomarkers or therapeutic targets in cancer patients and individuals with other conditions.^10^

Recently, circRNAs have been found to be dysregulated in HCC and to play an important functional role in this pathological context.^11^, ^12^, ^13^ The recently identified circRNA hsa_circ_0000615 has been found to play an oncogenic role in prostate,^14^ breast,^15^ gastric,^16^ and colorectal cancers.^17^ Moreover, hsa_circ_0000615 has been found to promote HCC cell migration, invasion, stemness, and proliferation.^18^, ^19^ How hsa_circ_0000615 functions in the context of tumor chemoresistance, however, remains to be defined.

Herein, we explored the expression of hsa_circ_0000615 in HCC patient serum and its relationship with patient clinical findings. Overall, we found that sorafenib‐resistant HCC patients exhibited hsa_circ_0000615 upregulation that was related to poorer overall survival (OS) outcomes. Moreover, hsa_circ_0000615 exhibited reasonably good area under the ROC curve (AUC) values, suggesting that it may offer value as a novel prognostic biomarker of sorafenib‐resistant HCC.

## 2 MATERIALS AND METHODS

### 2.1 Cell culture and clinical samples

Human Hep G2 and Huh 7 were grown in DMEM (Invitrogen, NY, USA). Hep G2/sorafenib and Huh 7/sorafenib cell lines were established by maintaining Hep G2 and Huh 7 cells at 1 mmol/L sorafenib and gradually increasing it at a rate of 0.5 mmol/L per month (up to 5 mmol/L) more than 10‐month. Serum samples from 202 HCC patients and 202 healthy controls were obtained from the First Affiliated Hospital of Bengbu Medical College. This study was approved by the Ethics Committee of the First Affiliated Hospital of Bengbu Medical College, with all patients having provided written informed consent.

### 2.2 Quantitative real‐time polymerase chain reaction

An RNA Isolation Kit (Vazyme Biotech, Nanjing, China) was used to extract total RNA from 500 μl of patient serum, after which a Prime Script RT reagent Kit (Takara, Dalian, China) was used for cDNA synthesis. Prepared cDNA was then used as input for qPCR reactions performed with SYBR Green (Takara). The U6 small nuclear B noncoding RNA (U6) was used to normalize expression values via the 2^−ΔΔCt^ method, with primers used being as follows: hsa_circ_0000615: F 5′–CAGCGCTATCCTTTGGGA–3′, R 5′–GACCTGCCACATTGGTCAGTA–3′; U6: F 5′–TGCGGGTGCTCGCTTCGGCAGC–3′, R 5′–GTGCAGGGTCCGAGGT–3′.

### 2.3 Statistical analysis

Data are means ± standard deviation (SD) and were compared via Student's *t* tests using GraphPad Prism 7. The Kaplan–Meier method was used for survival analyses, with *p* < .05 as the threshold of significance.

## 3 RESULTS

### 3.1 Hepatocellular carcinoma patients exhibit serum hsa_circ_0000615 upregulation

**FIGURE 1:**
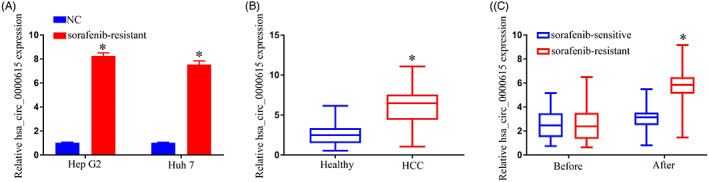
Hepatocellular carcinoma (HCC) patient serum samples exhibit hsa_circ_0000615 upregulation. (A). Hsa_circ_0000615 levels were significantly increased in sorafenib‐resistant HCC cells. (B). Serum hsa_circ_0000615 levels were higher in HCC patients. (C). sorafenib‐resistant patients (*n* = 122) exhibited higher levels of hsa_circ_0000615 expression compared with sorafenib‐sensitive patients in the before treatment and after treatment (*n* = 80). *p < .05.

We began by assessing the levels of hsa_circ_0000615 in control and sorafenib‐resistant HCC cells. The sorafenib‐resistant Hep G2/sorafenib and Huh 7/sorafenib cell lines exhibited marked upregulation of this circRNA relative to corresponding parental cell lines (Figure 1A). To explore the potential utility of hsa_circ_0000615 as a biomarker of chemoresistance, we then assessed the levels of this circRNA in serum samples from 202 HCC patients and 202 healthy controls. HCC patients exhibited significantly elevated serum hsa_circ_0000615 levels compared with healthy controls (Figure 1B). Moreover, hsa_circ_0000615 expression levels were higher in sorafenib‐resistant patients (*n* = 122) relative to those in sorafenib‐sensitive individuals (*n* = 80) (Figure 1C). As such, hsa_circ_0000615 offers potential value as an HCC chemotherapy biomarker.

### 3.2 Hsa_circ_0000615 levels are related to clinical features in HCC patients

**FIGURE 2:**
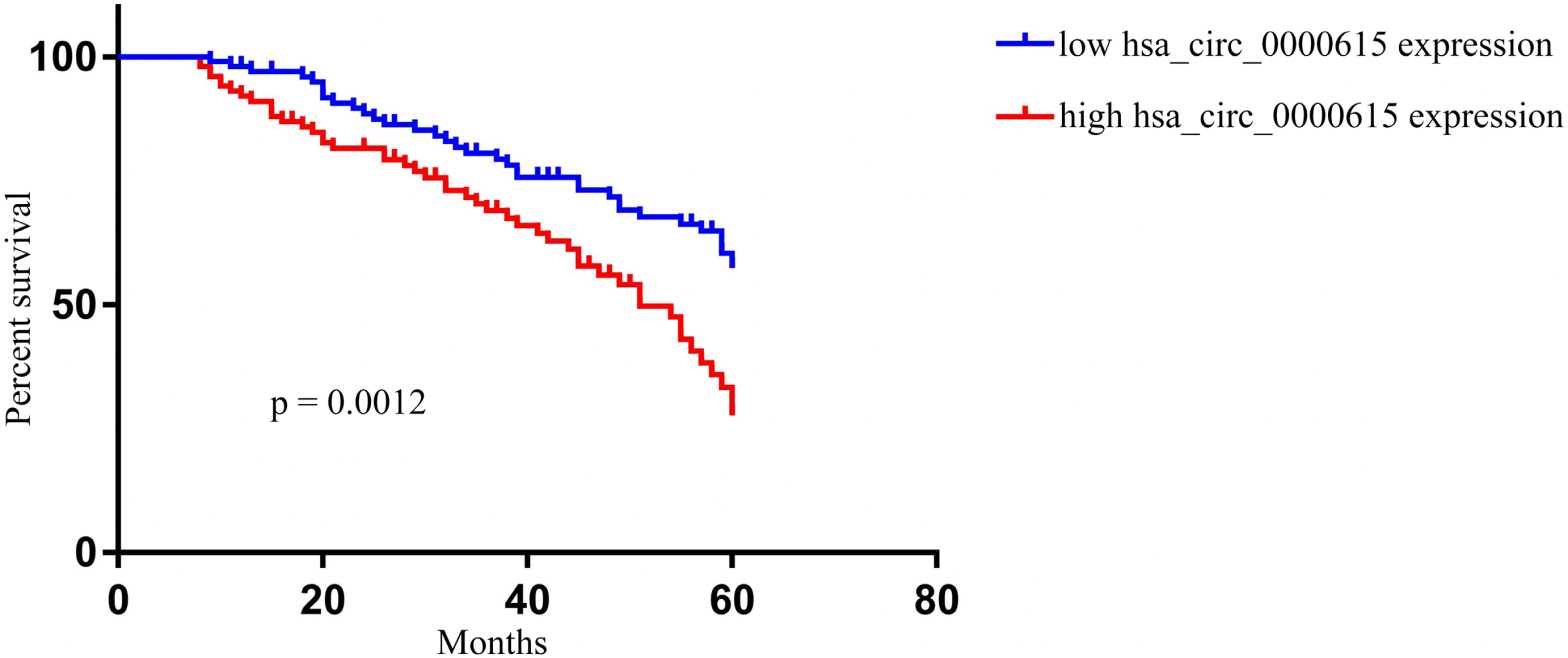
Hsa_circ_0000615 levels are linked with hepatocellular carcinoma patient survival. Patients exhibiting higher hsa_circ_0000615 expression levels exhibited prolonged overall survival compared with patients with lower levels of this circRNA.

Next, we stratified HCC patients into those with high and low levels of serum hsa_circ_0000615 based on the mean expression of this circRNA in this cohort and compared clinical features between these two patient groups. Chi‐squared analyses revealed that hsa_circ_0000615 expression was associated with clinical stage, TNM stage, and lymph node metastasis (Table 1), but was unrelated to age, sex, or histological grade. Kaplan–Meier analyses indicated that patients exhibiting higher levels of hsa_circ_0000615 expression presented with shorter OS relative to patients expressing low hsa_circ_0000615 levels (Figure 2).

**TABLE 1:** Correlations between hsa_circ_0000615 levels and hepatocellular carcinoma patient clinicopathological features

Characteristics	No.	hsa_circ_0000615 expression		*p*‐value
		High	Low	
Gender				
Male	112	53	59	.856
Female	90	48	42	
Age				
<60	108	55	53	.715
≥60	94	46	48	
TNM stage				
I‐II	81	59	22	<.05
III–IV	121	43	78	
Lymph node metastasis				
Positive	118	47	71	<.05
Negative	84	54	30	
Clinical stage				
I–II	93	61	32	<.05
III–IV	109	39	70	
Histological grade				
I	102	53	49	.642
II–III	100	48	52	

### 3.3 Hsa_circ_0000615 is associated with poor chemoresistant hepatocellular carcinoma patient prognosis

**FIGURE 3:**
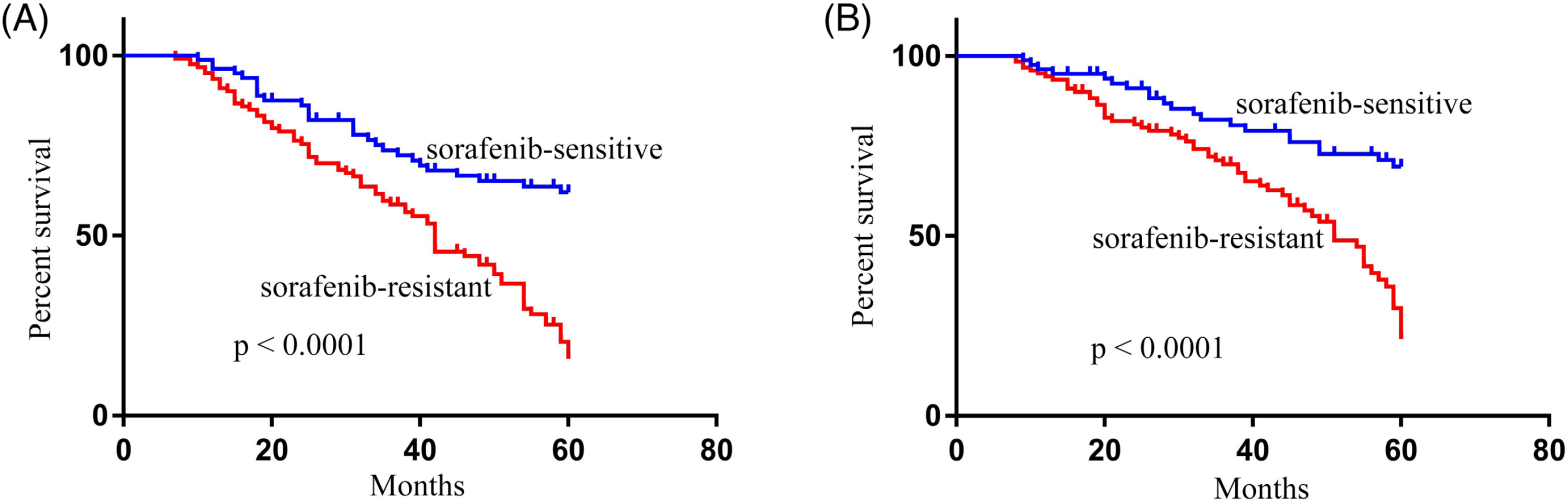
Hsa_circ_0000615 levels were significantly linked to poor outcomes in chemoresistant hepatocellular carcinoma (HCC) patients. Chemoresistant HCC patients exhibited significantly decreased progression‐free survival (A) and overall survival (B) relative to chemosensitive patients.

Through Kaplan–Meier analyses and log‐rank tests, we found chemoresistant HCC patients to exhibit significantly reduced OS and progression‐free survival (PFS) compared with chemosensitive patients (Figure 3). Through Cox proportional hazards regression analyses, we determined that clinical stage, chemoresistance, TNM stage, lymph node metastasis, and hsa_circ_0000615 levels were associated with patient PFS (Table 2) and OS (Table 3), highlighting hsa_circ_0000615 as a promising independent predictor of chemoresistant HCC patient survival.

**TABLE 2:** Univariate and multivariate analyses of hepatocellular carcinoma patient progression‐free survival

Variables	Univariate analysis		*p* value	Multivariate analysis		*p* value
	HR	95% CI		HR	95% CI	
Age	1.254	0.512–1.452	.354	‐	‐	‐
Gender	1.352	0.475–1.607	.248	‐	‐	‐
TNM stage	2.451	1.107–2.943	.004	2.254	0.925–2.719	.005
Lymph node metastasis	2.619	1.524–3.157	.003	2.146	1.159–2.943	.001
Clinical stage	3.521	1.432–4.025	.003	3.622	1.352–5.021	.002
Histological grade	1.351	0.842–1.691	.318	‐	‐	‐
chemoresistance	3.691	2.032–6.852	.001	3.312	2.452–5.721	.002
hsa_circ_0000615 expression	3.259	1.564–5.521	.002	3.157	1.425–4.917	.003

**TABLE 3:** Univariate and multivariate analyses of hepatocellular carcinoma patient overall survival

Variables	Univariate analysis		*p* value	Multivariate analysis		*p* value
	HR	95% CI		HR	95% CI	
Age	1.149	0.455–1.354	.319	‐	‐	‐
Gender	1.219	0.411–1.368	.223	‐	‐	‐
TNM stage	2.354	1.025–2.754	.005	2.157	0.856–2.654	.004
Lymph node metastasis	2.475	1.452–2.952	.004	2.241	1.242–2.815	.002
Clinical stage	3.145	1.325–3.954	.004	4.152	1.248–5.754	.001
Histological grade	1.222	0.658–1.254	.275	‐	‐	‐
chemoresistance	3.119	1.954–6.451	.001	2.975	1.956–4.595	.003
hsa_circ_0000615 expression	3.019	1.425–4.932	.004	2.842	1.322–4.571	.005

### 3.4 Serum hsa_circ_0000615 levels offer diagnostic utility for the detection of hepatocellular carcinoma chemoresistance

**FIGURE 4:**
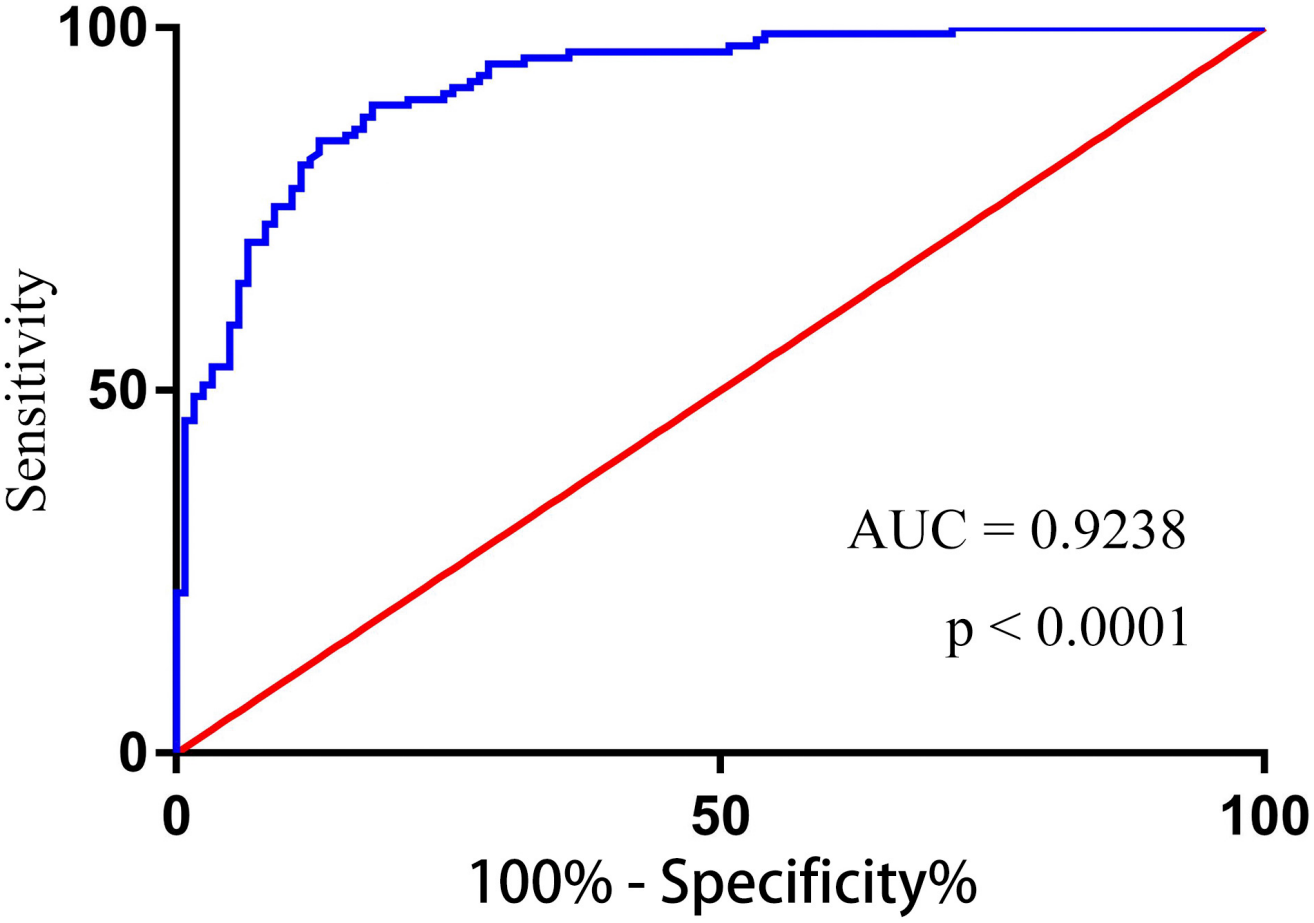
Serum hsa_circ_0000615 levels offer diagnostic value as predictors of hepatocellular carcinoma (HCC) patient chemoresistance. Receiver‐operating characteristic curves were used to differentiate between chemoresistant HCC patients before and after therapy.

Previous studies have shown that circRNAs show excellent potential diagnostic utility in various cancers, such as, breast cancer,^20^ gastric cancer,^21^ and HCC.^22^ To assess the potential diagnostic utility of serum hsa_circ_0000615 in patients with HCC, the area under the receiver operating characteristic (ROC) curve (AUC) was determined and found to be 0.9238 (95% CI, 0.8915–0.956, Figure 4, *p* < .0001), consistent with the value of serum hsa_circ_0000615 as a biomarker capable of differentiating between HCC patients and healthy controls.

## 4 DISCUSSION

Herein, we found that serum samples from HCC patients exhibited significant increases in hsa_circ_0000615 levels compared with those from control individuals. Moreover, the upregulation of this circRNA in samples from sorafenib‐resistant HCC patients relative to those from chemosensitive patients suggested that it may offer value as an independent predictor of patient outcomes.

A growing body of evidence suggests that circRNAs are functionally important in cancer and may offer value as predictive biomarkers or therapeutic targets. In colorectal cancer, for example, circRNA_0000392 can promote tumor progression via the miR‐193a‐5p/PIK3R3/AKT axis.^23^ Moreover, in non‐small cell lung cancer, circNDUFB2 can destabilize IGF2BPs and activate anti‐tumor immune responses to suppress tumor progression.^24^ In HCC, circRNA‐SORE can stabilize YBX1 to drive sorafenib resistance.^25^ As such, circRNAs function in a tumor‐specific manner.

Neoadjuvant chemotherapy is a mainstay of treatment for many cancers and has been used with increasing frequency over the past decade, with sorafenib‐based neoadjuvant chemotherapy being a standard of care for HCC patients. Those HCC patients that undergo sorafenib‐based chemotherapy prior to radical cystectomy exhibit better OS outcomes, but a subset of patients fail to attain any benefit from such treatment, with pathological responses to neoadjuvant chemotherapy being predictive of disease‐specific survival outcomes. Identifying reliable biomarkers capable of guiding clinicians to the selection of patients most likely to benefit from chemotherapeutic intervention is thus a critical clinical task.

In HCC, circRNAs can function as central regulators of sorafenib‐resistance,^26^, ^27^, ^28^ with hsa_circ_0000615 having previously been shown to drive HCC tumor growth and metastatic progression.^18^, ^19^ In this study, we further found hsa_circ_0000615 to be expressed at significantly higher levels in Hep G2/sorafenib and Huh 7/sorafenib cells relative to corresponding parental cell lines. The expression of this circRNA was similarly elevated in sorafenib‐resistant HCC patients compared with their chemosensitive counterparts, suggesting that hsa_circ_0000615 may offer value as a predictor of chemotherapeutic responses. Levels of hsa_circ_0000615 were also related to clinical stage, lymph node metastasis, and T stage in HCC patients, although they were unrelated to tumor histological stage, N stage, M stage, or patient age and gender. Kaplan–Meier analyses indicated that higher levels of hsa_circ_0000615 expression were associated with shorter patient OS compared with low levels of this circRNA. Moreover, chemoresistant HCC patients exhibited shorter OS and PFS compared with chemosensitive patients. Univariate and multivariate analyses further revealed clinical stage, T stage, lymph node metastasis, and chemoresistance to be correlated with OS and PFS outcomes, suggesting hsa_circ_0000615 to be a valuable independent predictor of HCC patient outcomes. In addition, the AUC value for this circRNA in HCC patients was 0.9238, indicating that serum levels of hsa_circ_0000615 can be used to reliably differentiate between HCC patients and healthy individuals.

## 5 CONCLUSIONS

In summary, we herein found hsa_circ_0000615 upregulation to be prominent within serum samples from HCC patients, with such upregulation being significantly more pronounced in samples from chemosensitive patients relative to chemoresistant patients. As such, hsa_circ_0000615 is a promising target that warrants further study in an effort to understand the mechanistic basis for HCC patient chemoresistance.

## CONFLICT OF INTEREST

The authors of this work declare that they have no conflict of interest.

## Data Availability

Due to the nature of this research, participants of this study did not agree for their data to be shared publicly, so supporting data are not available.
